# Colonic epithelial cathelicidin (LL‐37) expression intensity is associated with progression of colorectal cancer and presence of CD8 ^+^ T cell infiltrate

**DOI:** 10.1002/cjp2.222

**Published:** 2021-05-14

**Authors:** Ross J Porter, Graeme I Murray, Abdo Alnabulsi, Matthew P Humphries, Jacqueline A James, Manuel Salto‐Tellez, Stephanie G Craig, Ji M Wang, Teizo Yoshimura, Mairi H McLean

**Affiliations:** ^1^ Centre for Inflammation Research, Queens Medical Research Institute University of Edinburgh Edinburgh UK; ^2^ School of Medicine, Medical Sciences and Nutrition University of Aberdeen Aberdeen UK; ^3^ Precision Medicine Centre of Excellence, The Patrick G Johnston Centre for Cancer Research Queen's University Belfast UK; ^4^ Integrated Pathology Programme, Division of Molecular Pathology The Institute of Cancer Research London UK; ^5^ Cancer and Inflammation Program, Center for Cancer Research National Cancer Institute at Frederick Frederick MD USA; ^6^ Department of Pathology and Experimental Medicine, Graduate School of Medicine, Dentistry and Pharmaceutical Sciences Okayama University Okayama Japan; ^7^ Division of Molecular & Clinical Medicine, School of Medicine University of Dundee Dundee UK

**Keywords:** colorectal cancer, LL‐37, cathelicidin, organoid, lymphocytes

## Abstract

Colorectal cancer (CRC) remains a leading cause of cancer mortality. Here, we define the colonic epithelial expression of cathelicidin (LL‐37) in CRC. Cathelicidin exerts pleotropic effects including anti‐microbial and immunoregulatory functions. Genetic knockout of cathelicidin led to increased size and number of colorectal tumours in the azoxymethane‐induced murine model of CRC. We aimed to translate this to human disease. The expression of LL‐37 in a large (*n* = 650) fully characterised cohort of treatment‐naïve primary human colorectal tumours and 50 matched normal mucosa samples with associated clinical and pathological data (patient age, gender, tumour site, tumour stage [UICC], presence or absence of extra‐mural vascular invasion, tumour differentiation, mismatch repair protein status, and survival to 18 years) was assessed by immunohistochemistry. The biological consequences of LL‐37 expression on the epithelial barrier and immune cell phenotype were assessed using targeted quantitative PCR gene expression of epithelial permeability (*CLDN2*, *CLDN4*, *OCLN*, *CDH1*, and *TJP1*) and cytokine (*IL‐1β*, *IL‐18*, *IL‐33*, *IL‐10*, *IL‐22*, and *IL‐27*) genes in a human colon organoid model, and CD3^+^, CD4^+^, and CD8^+^ lymphocyte phenotyping by immunohistochemistry, respectively. Our data reveal that loss of cathelicidin is associated with human CRC progression, with a switch in expression intensity an early feature of CRC. LL‐37 expression intensity is associated with CD8^+^ T cell infiltrate, influenced by tumour characteristics including mismatch repair protein status. There was no effect on epithelial barrier gene expression. These data offer novel insights into the contribution of LL‐37 to the pathogenesis of CRC and as a therapeutic molecule.

## Introduction

Human cathelicidin (and its bioactive derivative LL‐37) is an anti‐microbial peptide, expressed by epithelial cells at mucosal surfaces and a wide array of immune cells [1]. LL‐37 is anti‐microbial, acts as a chemoattractant, impacts innate and adaptive immune cell responses, contributes to host–microbiome interaction, and has a role in wound healing [2, 3]. Cathelicidin plays a role in the pathogenesis of malignancy and, given its pleotropic biological responses, can exert pro‐malignant or anti‐tumoural effects and this is dependent on tissue type [2, 4]. In the gut, genetic knockout of cathelicidin (CRAMP in the mouse) led to shorter crypt length implying a homeostatic role in colonic epithelial growth dynamics [5]. Presence of cathelicidin is associated with a robust mucus layer in structure and function [6, 7]. There is evidence to suggest a protective role of cathelicidin in colonic carcinogenesis through effects on growth dynamics, particularly apoptosis and autophagy, angiogenesis, and fibroblast activity [1, 2, 8] and linked to the colonic microbiome [5]. Our previous data revealed that genetic knockout of CRAMP (murine cathelicidin) led to increased tumour size and number in the azoxymethane‐induced murine model of colorectal cancer (CRC), suggesting a tumour‐suppressive role [5].

CRC remains a leading cause of cancer in the UK and worldwide [9]. Despite advances in treatments and earlier detection through national screening programmes, many people still die from this malignancy. CRC is ranked as the second most lethal cancer, accounting for 9.2% of cancer mortality [9]. Therefore, there is a clinical need to increase understanding of the pathogenesis of this malignancy and to develop prognostic biomarkers and new treatment targets.

The pathogenesis of CRC is multifactorial and involves a series of genetic and epigenetic mutations, microbial dysbiosis, intestinal inflammation, and epithelial barrier dysfunction [10, 11, 12].

The key question now is whether the protective role for LL‐37 in our pre‐clinical studies translates to human disease. Evidence for this is limited. Ren *et al* [13] characterised cathelicidin expression in a cohort of 102 human CRCs and demonstrated a reduced expression compared to normal colon tissue.

This study aimed to define the epithelial cytoplasmic expression profile of cathelicidin linked to clinico‐pathological factors in human CRC, assess whether this could be a biomarker for prognosis, and explore the biological consequences of expression on the epithelial barrier and immune cell phenotype.

We now demonstrate the expression of LL‐37 in a large (*n* = 650) fully characterised cohort of treatment‐naïve primary human colorectal tumours and 50 matched normal mucosa samples and show expression intensity of epithelial cathelicidin is associated with human CRC progression and CD8^+^ T cell infiltrate.

## Materials and methods

### Ethical approval

Ethical approval for use of human tissue and data in this study was obtained from the Scientific Access Committee of the Grampian Tissue Biorepository (Tissue Request No. 000135 for Tissue Microarray and 000176 for organoid culture). The biorepository has delegated research ethics authority (11/NS/0015) from The North of Scotland Research Ethics Committee to approve research projects involving human tissue and data. All tissue and data were anonymised. Project specific written consent was not required for the retrospective use of archival tissue. Patients provided written consent prior to surgery for the use of their resected tissue for research purposes to the Grampian Tissue Biorepository and this was subsequently used for the organoid culture model.

### Colorectal cancer tissue microarray

Formalin‐fixed paraffin‐embedded tissue cores from 650 CRCs and 50 matched normal pairs were presented within a previously published well‐characterised tissue microarray (TMA) with long‐term overall survival data [14, 15, 16]. Tissue cores were obtained from chemotherapy and radiotherapy‐naïve patients undergoing elective surgery for primary CRC between 1994 and 2009 at Aberdeen Royal Infirmary, Scotland, UK. Each tumour was histologically reported according to the Royal Collage of Pathologists UK guidelines, and represented by two 1 mm cores on the tissue microarray. The area of the tumour to be sampled for microarray construction was selected by an expert gastrointestinal pathologist (GIM) based on histological assessment of a formalin‐fixed paraffin‐embedded section of the tumour. The normal mucosal samples were retrieved from the resection margin, from an area at least 10 cm distant from the tumour. The relationship between clinico‐pathological parameters and overall survival validates the tissue microarray as representative of pathology (Table 1).

**Table 1:** CRC tissue microarray with clinico‐pathological data.

Characteristic	No. of patients	Percentage	Relationship with survival
Gender			
Male	340	52.3	*χ*^2^ = 0.027, *p* = 0.870
Female	310	47.7	
Age			
<70	305	46.9	*χ*^2^ = 29.213, ***p* < 0.001**
≥70	345	53.1	
Screening detected			
Yes	52	8	*χ*^2^ = 16.381, ***p* < 0.001**
No	598	92	
Tumour site			
Proximal colon	261	40.2	Proximal versus distal, *χ* ^2^ = 8.418, ***p* = 0.004**
Distal colon	245	37.7	Distal versus rectal, *χ* ^2^ = 0.906, *p* = 0.341
Rectum	144	22.2	Colon versus rectum, *χ* ^2^ = 0.098, *p* = 0.754
Tumour differentiation			
Well/moderate	600	92.3	*χ*^2^ = 0.976, *p* = 0.323
Poor	50	7.7	
Extra‐mural venous invasion (EMVI)			
Present	140	21.5	*χ*^2^ = 100.946, ***p* < 0.001**
Absent	510	78.5	
Mismatch repair protein status (defined by MLH1 and MSH2 immunohistochemistry)			
Deficient	96	15.2	*χ*^2^ = 2.848, *p* = 0.091
Proficient	536	84.8	
pT stage			
T1	30	4.6	T1 versus T2, *χ* ^2^ = 0.382, *p* = 0.536
T2	114	17.5	T2 versus T3, *χ* ^2^ = 24.739, ***p* < 0.001**
T3	411	63.2	T3 versus T4, *χ* ^2^ = 30.159, ***p* < 0.001**
T4	95	14.6	
pN stage			
N0	364	56	N0 versus N1, *χ* ^2^ = 54.071, ***p* < 0.001**
N1	177	27.2	N1 versus N2, *χ* ^2^ = 17.636, ***p* < 0.001**
N2	109	16.8	
The Union for International Cancer Control (UICC) stage			
1	120	18.5	A versus B, *χ* ^2^ = 5.059, ***p* = 0.025**
2	244	37.5	B versus C, *χ* ^2^ = 65.510, ***p* < 0.001**
3	286	44	

Analysis with chi‐square tests validates the TMA as representative of pathology. Values in bold indicate *p* < 0.05.

### Clinical and pathological data

Clinical and pathological data were available for each case represented on the tissue microarray as described in Table 1. Clinical data included survival up to 18.2 years (mean 5.5 years, SD 3.8), patient age at resection and gender, alongside tumour characteristics such as tumour site and stage [The Union for International Cancer Control (UICC)] as a composite score from tumour stage and nodal status). Pathological data included presence or absence of extra‐mural vascular invasion (EMVI), tumour differentiation, and mismatch repair protein status defined by proficient or deficient MLH1 and MSH2 status determined by immunohistochemistry.

### Immunohistochemistry

Intensity of epithelial cytoplasmic LL‐37 was assessed by immunohistochemistry using rabbit anti‐human cathelicidin antibody (ab69484; Abcam, Cambridge, UK) at a dilution of 1:200. Tissue sections of 4 μm were cut and placed onto 3‐aminopropyltriethoxysilane‐coated slides for immunohistochemical analysis. Specimens were dewaxed in xylene and rehydrated in alcohol. Antigen retrieval was performed in EDTA (pH 7.8) buffer for 20 min. EnVision+™ peroxidase‐linked, biotin‐free synthesis (Dako, Ely, UK) with 3'‐3'‐diaminobenzidine as chromogen was used for detection using a Dako Autostainer (Dako), as previously published [14, 15, 16, 17]. Sections were counterstained with haematoxylin. Primary antibody was applied to the tissue sections and incubated for 60 min within the staining protocol. A negative (omitting primary antibody) and positive control (human spleen) were included.

The immunohistochemical assessment of lymphocyte infiltrate (CD3^+^, CD4^+^, and CD8^+^ cells) was performed as part of a different study as previously published [18], and the raw data were obtained through the Grampian Biorepository for this new analysis. In summary, CD3^+^ and CD4^+^ cells were detected using the Ventana Benchmark XT autostainer (Ventana Medical Systems, Oro Valley, AZ, USA), whereas CD8 was detected using the Leica autostainer (Leica Biosystems, Wetzlar, Germany) [18]. The immunostaining was completed using CONFIRM Anti‐CD3 (clone: 2GV6) and CONFIRM anti‐CD4 (clone: SP35) neat primary rabbit monoclonal antibodies (Ventana Medical Systems), and anti‐CD8 (clone: CD/144B) primary mouse antibody at a dilution of 1:50 (Agilent Technologies, Santa Clara, CA, USA). Antigen retrieval was performed using Cell Conditioning Solution (CC1) buffer (Ventana Medical Systems) for 32 and 60 min for anti‐CD3 and anti‐CD4, respectively. Antigen retrieval for anti‐CD8 was completed using ER2 solution (Leica Biosystems) for 20 min. Primary antibodies were applied for 16 min at 37°C for anti‐CD3 and anti‐CD4, and 15 min at room temperature for anti‐CD8 [18]. A negative (omitting primary antibody) and positive control (human tonsil) were included.

### Scoring of LL‐37 expression

Expression intensity of epithelial cytoplasmic LL‐37 was independently assessed by two observers (RJP and MHM), blinded to clinical and pathological data, as absent, weak, moderate, or strong by light microscopy as previously published [14, 15, 16, 17]. LL‐37 expression was heterogeneous across the cores and therefore we applied the criteria of reporting on the area of most positive expression intensity and this was applied to all cores to ensure uniformity of approach. Any discrepancies in the scores were resolved through simultaneous re‐evaluation of the cores by both observers, and review by an expert gastrointestinal pathologist (GIM). Cases were recorded as missing if the evaluation of immunostaining was not possible due to the tissue cores being damaged/folded during the tissue preparation process or there were no tumour cells present in the core.

### Scoring of immune cell phenotype

All slides immunostained for immune cell infiltrate were scanned at ×40 magnification using a Leica Aperio AT2 whole slide scanner and digital image analysis conducted as previously published [18]. In brief, immunostaining of all immune cell biomarkers was scored using QuPath software version 0.1.2 [19]. The cores were first annotated with the assistance of a senior consultant pathologist before applying an automated scoring algorithm for each immune biomarker [18]. Cases were recorded as missing if the evaluation of immunostaining was not possible due to the tissue cores being damaged or folded. Immune cell results were quantified according to average density of positive cells present for all TMA cores available for each patient. These results were then ranked by increasing density and four equal groups representing increasing relative immune cell density within the patient cohort were generated in order to create immune phenotype data comparable to the data generated for LL‐37 expression groups.

### Human colon‐derived epithelial organoid model

A human colon‐derived epithelial organoid model was established from fresh colon tissue, optimised at our centre from a previously published protocol [20]. A 3–5‐cm^3^ piece of non‐neoplastic colonic mucosa, dissected from the resection margin, was washed in cold DPBS, cut into 5–10 mm pieces, and chelated in 2.5 mm EDTA buffer on ice for 40 min with gentle agitation. Tissue was shaken vigorously to liberate crypts and passed through a 100‐μm cell strainer. Crypt containing elute was centrifuged at 4 °C at 300 × *g* for 3 min and the pellet was resuspended in cold DPBS and washed three times to remove single‐cell debris. Crypts were suspended in Matrigel© (Corning, NY, USA), supplemented with 1:100 Jagged‐1‐peptide, at 200 crypts/15 μl Matrigel©. Crypt‐Matrigel© mixture/well of 15 μl was dispensed into a pre‐warmed delta‐surface 24‐well plate, and Matrigel© polymerised by inverting plate at 37°C for 15 min. For initiating, maintaining, and differentiation of colonic organoid culture, a 50% conditioned media (1:1 dilution in advanced DMEM/F12 base media) was used, generated from genetically modified L‐WRN mouse fibroblast cell line (CLR‐3276; ATCC, Manassas, VA, USA) cultured in advanced D‐MEM/F12 with 10% FCS, 0.5 mg/ml hygromycin‐B, and 0.5 mg/ml G‐418. This cell line secretes human Wnt3A, R‐spondin, and noggin, essential factors for intestinal epithelial cell organoid culture. Additional components of the initiation media are listed in Supplementary materials and methods. Organoids were cultured at 37 °C in 5% CO_2_. After 48 h, initiation media was replaced with maintenance media (see Supplementary materials and methods) and this was replaced every 3–4 days. Before experimental stimulations, organoids were plated on delta‐surface 96‐well plates and cultured in fully recombinant differentiation media (see Supplementary materials and methods) for at least 3–4 days to maturity.

### Epithelial permeability and cytokine gene expression profile

RNA was extracted from organoid cultures (*n* = 3 individual patients with 3 technical replicates/experiment) using RNeasy RNA isolation kit (Qiagen, Hilden, Germany) and quantified by Nanodrop spectroscopy. RT‐qPCR using TaqMan assays (Applied Biosystems, Foster City, CA, USA) characterised organoid permeability (*CLDN2*, *CLDN4*, *OCLN*, *CDH1*, and *TJP1*) and epithelial‐derived cytokine (*IL‐1β*, *IL‐18*, *IL‐33*, *IL‐10*, *IL‐22*, and *IL‐27*) gene expression normalised to *GAPDH* and *B2M* following a 48‐h stimulation with recombinant LL‐37 (TOCRIS 154947‐66‐7, Tocris Bioscience, Avonmouth, UK) at 0 and 100 ng/ml. Relative gene expression was calculated using the Livak method [21].

### Data analysis

Data were analysed with IBM®SPSS® Version 25.0 (IBM, Portsmouth, UK) and Prism Version 8 (GraphPad Software Inc., San Diego, CA, USA). Statistical tests were Pearson's chi‐square test, Fisher's exact test, ANOVA, and log‐rank test with Kaplan–Meier survival analysis, as appropriate. A two‐tailed alpha was set at 0.05 and 95% CIs were included where appropriate. Scores for LL‐37 expression were also dichotomised using negative versus positive staining, negative and weak staining versus moderate and strong staining, and strong versus negative/weak/ moderate staining comparisons as previously published [14, 15, 16, 17]. For survival analysis and for comparison with LL‐37, the scores of immune cell biomarkers were (1) dichotomised based on quartiles into four different categories (0, 1, 2, and 3 representing 0–25, 26–50, 51–75, and 76–100% density as a percentage of total immune cell density, respectively) and (2) assessed as continuous data defined as the number of immune cells per core. All analyses were performed without imputation of missing values.

## Results

**Figure 1:**
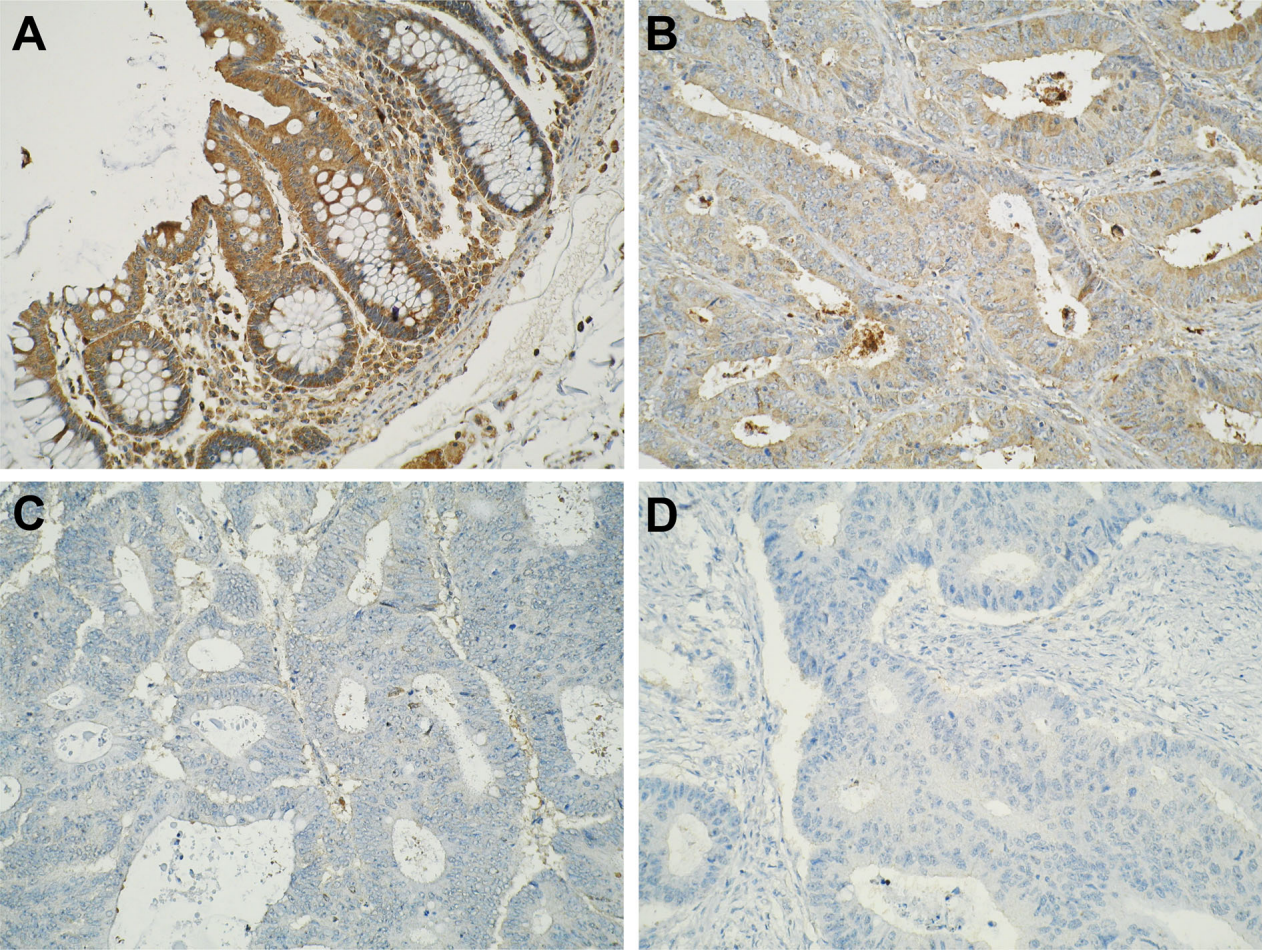
Representative photomicrographs of epithelial cytoplasmic LL‐37 expression intensity in (A) normal colonic mucosa, (B) UICC stage 1 CRC, (C) UICC stage 2 CRC, and (D) UICC stage 3 CRC. The digital photographs were taken using an Olympus BX51 microscope with a digital Olympus camera (Olympus, Tokyo, Japan), with objective magnification of ×20.

Representative photomicrographs of epithelial cytoplasmic LL‐37 expression intensity across colorectal neoplastic progression are shown in Figure 1. In terms of interobserver variability of LL‐37 expression intensity, considering full assessment of cores (−1 [no epithelial component], 0 [absent], 1 [weak], 2 [moderate], and 3 [strong]), Cohen's kappa was 0.848 (95% CI 0.824, 0.872; *p* < 0.001). When considering scores that either assessor scored 0, 1, 2, and 3, the weighted kappa was 0.865 (95% CI 0.842, 0.887; *p* < 0.001). In both scenarios, a score of 0.81–1.00 indicates a satisfactory agreement between observers.

### Loss of LL‐37 expression in colonic epithelial cells is associated with CRC

**Figure 2:**
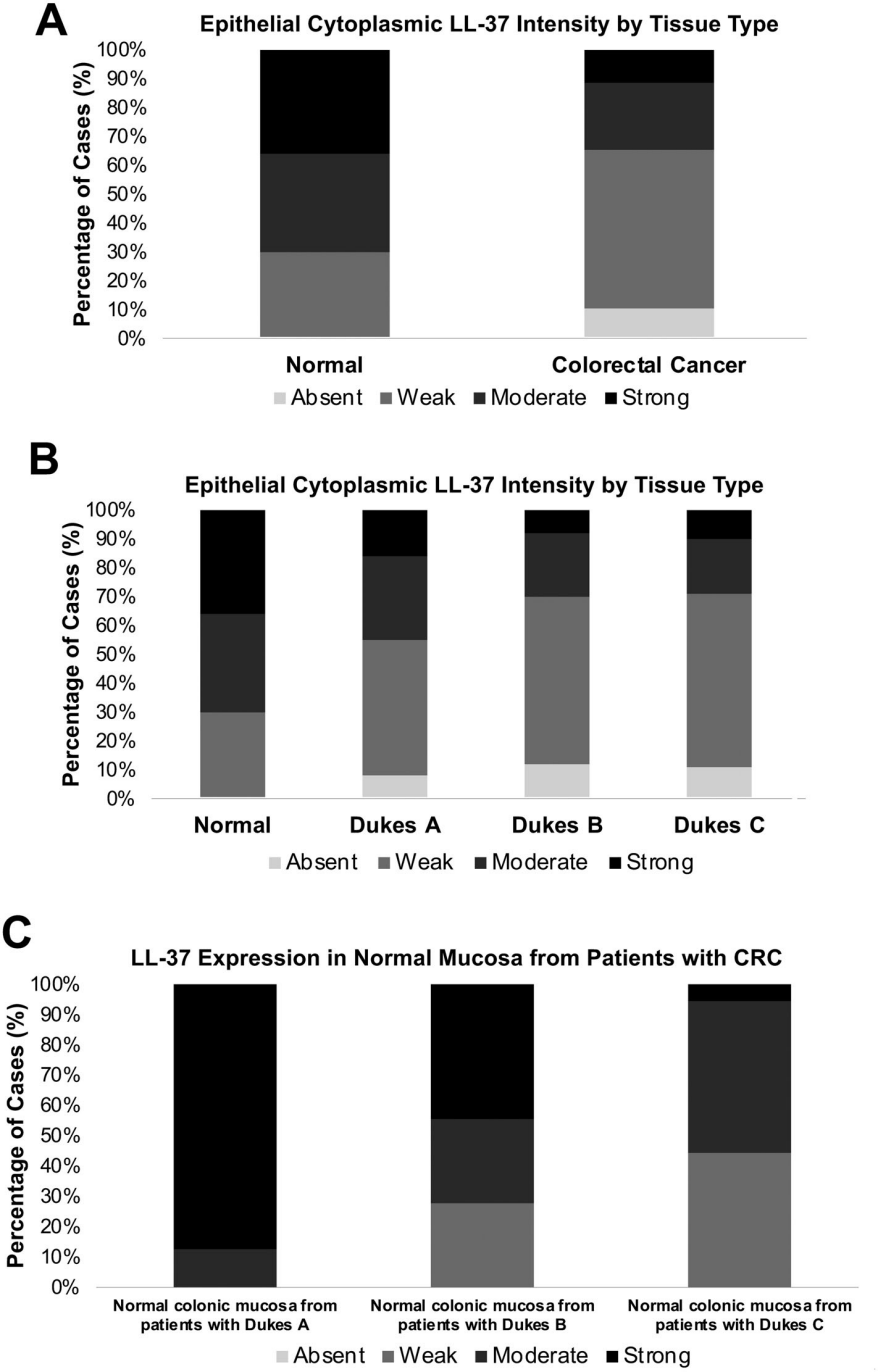
Frequency analysis of epithelial cytoplasmic LL‐37 expression intensity in normal and CRC tissue. (A) CRC tissue has reduced expression intensity of LL‐37 compared with normal tissue. (B) Loss of LL‐37 expression intensity occurs during the early stages of colorectal carcinogenesis. (C) Differential epithelial cytoplasmic LL‐37 expression intensity in non‐neoplastic colorectal epithelium adjacent to CRCs of different stage.

All non‐neoplastic normal tissue expressed LL‐37 in the cytoplasm of epithelial cells, with 30% (13/44), 34% (15/44), and 36% (16/44) expressing weak, moderate, and strong intensity, respectively. Expression intensity of LL‐37 was significantly reduced in CRC (absent versus weak versus moderate versus strong expression intensity, *p* < 0.001) (Figure 2A and Table 2, row 1). Of 589 CRC cores, 63 (11%) did not express LL‐37. Of those expressing epithelial cytoplasmic LL‐37 (*n* = 526), the majority displayed weak intensity expression (*n* = 332/526, 63%).

**Table 2:** Association between LL‐37 expression intensity and histological diagnosis.

Cytoplasmic epithelial LL‐37 comparisons	Absent versus weak versus moderate versus strong LL‐37		Absent versus weak, moderate, and strong LL‐37		Absent and weak versus moderate and strong LL‐37		Strong versus absent, weak, and moderate LL‐37	
	*χ* ^2^	*p*	*χ* ^2^	*p*	*χ* ^2^	*p*	*χ* ^2^	*p*
Normal versus CRC	30.191	**<0.001**	–	**0.016**	25.153	**<0.001**	25.298	**<0.001**
Normal versus UICC stage 1	11.679	**0.009**	–	0.064	7.845	**0.005**	7.455	**0.010**
Normal versus UICC stage 2	36.916	**<0.001**	–	**0.012**	26.071	**<0.001**	27.158	**<0.001**
Normal versus UICC stage 3	32.600	**<0.001**	–	**0.019**	26.985	**<0.001**	21.108	**<0.001**
UICC stage 1 versus 2 versus 3	12.727	**0.048**	1.690	0.430	10.639	**0.005**	5.879	0.053
UICC stage 1 versus UICC stage 2	10.007	**0.019**	1.671	0.196	8.323	**0.004**	5.698	**0.017**
UICC stage 2 versus UICC stage 3	1.414	0.706	0.364	0.566	0.004	1.000	0.712	0.399
UICC stage 1 versus UICC stage 3	8.967	**0.030**	0.713	0.398	9.910	**0.003**	2.853	0.091
UICC stage 1 versus UICC stages 2 and 3	11.403	**0.010**	1.305	0.253	10.635	**0.001**	5.254	**0.022**
UICC stages 1 and 2 versus UICC stage 3	2.504	0.475	0.007	0.933	2.035	0.154	0.059	0.808
Normal from UICC stage 1 versus normal from UICC stage 2 versus normal from UICC stage 3	16.930	**0.001**	–	–	5.301	0.090	16.931	**<0.001**

Analysed by chi‐square tests. Values in bold indicate *p* < 0.05.

### Loss of LL‐37 expression intensity is associated with CRC progression

The expression intensity of LL‐37 significantly decreased with increasing UICC stage (*p* = 0.048) (Figure 2B and Table 2). Specifically, our data reveal that the switch in expression occurs at the early stages of colonic carcinogenesis. Expression of LL‐37 was significantly lower in UICC stage 1 (*p* = 0.009), UICC stage 2 (*p* < 0.001), and UICC stage 3 (*p* < 0.001) tumours compared to normal. There was a significant reduction in LL‐37 intensity in UICC stage 2 (*p* = 0.019) and UICC stage 3 (*p* = 0.030) compared to UICC stage 1 tumours. There was no difference in LL‐37 expression intensity between UICC stage 2 and UICC stage 3 tumours (*p* = 0.706).

### The expression of LL‐37 in adjacent non‐neoplastic epithelium differs with tumour stage

There was a significant difference in epithelial LL‐37 expression intensity between non‐neoplastic normal colonic mucosa from patients with UICC stage 1, UICC stage 2, and UICC stage 3 CRC (*p* = 0.001) (Figure 2C and Table 2).

### Epithelial LL‐37 expression is not associated with survival in CRC

Next, we assessed the expression of LL‐37 in association with clinical and pathological parameters. The epithelial expression of LL‐37 was not associated with survival across the whole tissue cohort in univariate analysis (Table 3 and supplementary material, Figure S1A). Subgroup analysis of the different clinical and pathological features in isolation, such as tumour stage (UICC stage 1–3), did not reveal association with survival and LL‐37 expression (data not included). Multivariate analysis did not identify any association when factors such as age or sex were accounted for (see supplementary material, Figure S1B).

**Table 3:** Association between LL‐37 expression intensity and clinico‐pathological characteristics.

Cytoplasmic epithelial LL‐37 comparisons	Absent versus weak versus moderate versus strong LL‐37		Absent versus weak, moderate, and strong LL‐37		Absent and weak versus moderate and strong LL‐37		Strong versus absent, weak, and moderate LL‐37	
	*χ* ^2^	*p*	*χ* ^2^	*p*	*χ* ^2^	*p*	*χ* ^2^	*p*
Gender	2.393	0.495	0.811	0.368	1.200	0.273	0.051	0.821
Age	6.250	0.100	0.034	0.853	2.642	0.104	0.723	0.395
Screen detected	2.986	0.392	–	0.459	0.141	0.747	–	0.211
Tumour site	9.709	0.137	7.132	**0.028**	0.080	0.961	2.196	0.334
Colon versus rectum	4.695	0.196	3.536	0.060	0.010	1.000	0.869	0.351
Tumour differentiation	2.471	0.479	–	0.565	2.042	0.153	–	0.388
Extra‐mural venous invasion	3.065	0.383	1.139	0.286	0.683	0.409	0.097	0.755
Mismatch repair protein status	2.411	0.492	0.181	0.671	0.026	0.871	1.500	0.221
pT stage	15.137	0.087	2.521	0.471	7.005	0.072	8.824	**0.032**
pN stage	8.217	0.223	0.860	0.651	5.244	0.073	2.371	0.306
UICC stage	12.727	**0.048**	1.690	0.430	10.639	**0.005**	5.879	0.053
Survival	3.606	0.307	0.796	0.372	0.862	0.353	0.834	0.361

Parameters were analysed by chi‐square tests and survival was analysed by Kaplan–Meier (log‐rank) test. Values in bold indicate *p* < 0.05.

### Epithelial LL‐37 expression is associated with tumour site but no other clinical or pathological characteristics of CRC

Negative versus positive LL‐37 expression was associated with tumour site (*p* = 0.028); positive expression was especially associated with the proximal colon. Otherwise, we did not identify a significant association with any additional clinical or pathological factors (Table 3).

### Density of T cell infiltrate is associated with survival in CRC

As expected, survival across the tissue microarray was associated with T cell infiltrate (see supplementary material, Figure S2). The relationship between the density of T cell infiltrate (CD3^+^, CD4^+^, and CD8^+^ cells) and overall survival validates the tissue microarray as representative of pathology [18]. Representative photomicrographs of T cell infiltrate are published previously [18].

### Density of CD8 ^+^, but not CD3 ^+^ or CD4 ^+^, T cell infiltrate is associated with LL‐37 expression intensity

The density of CD8^+^ T cell infiltrate was associated with LL‐37 expression across the microarray (Table 4), and particularly absence versus presence of CD8^+^ cells with increasing expression intensity of LL‐37 (*p* = 0.002). This association was not seen for CD3^+^ or CD4^+^ cells (Table 4). To understand this relationship further, an analysis using continuous data of the number of infiltrating CD8^+^ immune cells revealed that LL‐37 expression is associated with the number of CD8^+^ T cells in CRC (*p* = 0.003) (see supplementary material, Figure S3): absent (215.43), weak (275.45), moderate (285.33), and strong (220.98) mean ranks suggest increased CD8^+^ cell infiltration from absent through moderate LL‐37 expression intensity and then reduced CD8^+^ cell infiltration with strong LL‐37 expression. We next assessed the continuous data of number of CD8^+^ cells in association with LL‐37 expression and other tumour characteristics. The relationship between LL‐37 expression and CD8^+^ T cells was observed in colonic tumour site (*p* = 0.012), moderate‐to‐well differentiation (*p* ≤ 0.005), absence of EMVI (*p* ≤ 0.038), and UICC stage 3 tumour stage (*p* ≤ 0.022) (see supplementary material, Table S1A–F). Notably, the association between CD8^+^ cell infiltrate and LL‐37 expression was more pronounced according to mismatch repair protein status, particularly in mismatch repair proficient tumours (*p* ≤ 0.036) (see supplementary material, Table S1F).

**Table 4:** Association between epithelial cell LL‐37 expression intensity (absent, weak, moderate, or strong) and density of T cell infiltrate.

	Absent versus weak versus moderate versus strong LL‐37		Absent versus weak, moderate, and strong LL‐37		Absent and weak versus moderate and strong LL‐37		Strong versus absent, weak, and moderate LL‐37	
	*χ* ^2^	*p*	*χ* ^2^	*p*	*χ* ^2^	*p*	*χ* ^2^	*p*
CD3+ T cells								
0 versus 1 versus 2 versus 3	6.294	0.710	1.435	0.697	1.545	0.672	2.718	0.437
0 and 1 versus 2 and 3	3.603	0.308	0.820	0.365	0.034	0.853	2.238	0.135
0 versus 1, 2, and 3	1.346	0.718	0.001	0.972	0.663	0.415	0.082	0.775
3 versus 0, 1, and 2	2.152	0.542	0.717	0.397	0.097	0.755	0.783	0.376
CD4+ T cells								
0 versus 1 versus 2 versus 3	8.709	0.465	3.912	0.271	2.049	0.562	2.237	0.525
0 and 1 versus 2 and 3	5.321	0.150	3.024	0.082	0.127	0.721	1.337	0.248
0 versus 1, 2, and 3	2.294	0.514	0.463	0.496	1.760	0.185	0.006	0.937
3 versus 0, 1, and 2	0.755	0.860	0.097	0.755	0.019	0.890	0.414	0.520
CD8+ T cells								
0 versus 1 versus 2 versus 3	17.083	**0.047**	8.051	**0.045**	0.011	1.000	6.704	0.082
0 and 1 versus 2 and 3	10.934	**0.012**	5.349	**0.021**	0.004	0.950	4.110	**0.043**
0 versus 1, 2, and 3	14.908	**0.002**	7.069	**0.008**	0.011	0.915	5.829	**0.016**
3 versus 0, 1, and 2	4.480	0.214	1.177	0.278	0.001	0.970	2.542	0.111

Analysed using categorical T cell infiltrate data (quartiles expressed as % of total immune cell density; 0 = 0–25%, 1 = 26–50%, 2 = 51–75%, and 3 = 76–100%) with chi‐square tests. Values in bold indicate *p* < 0.05.

### LL‐37 does not impact expression of epithelial barrier permeability or epithelial‐derived cytokine genes in a human colon‐derived organoid model

**Figure 3:**
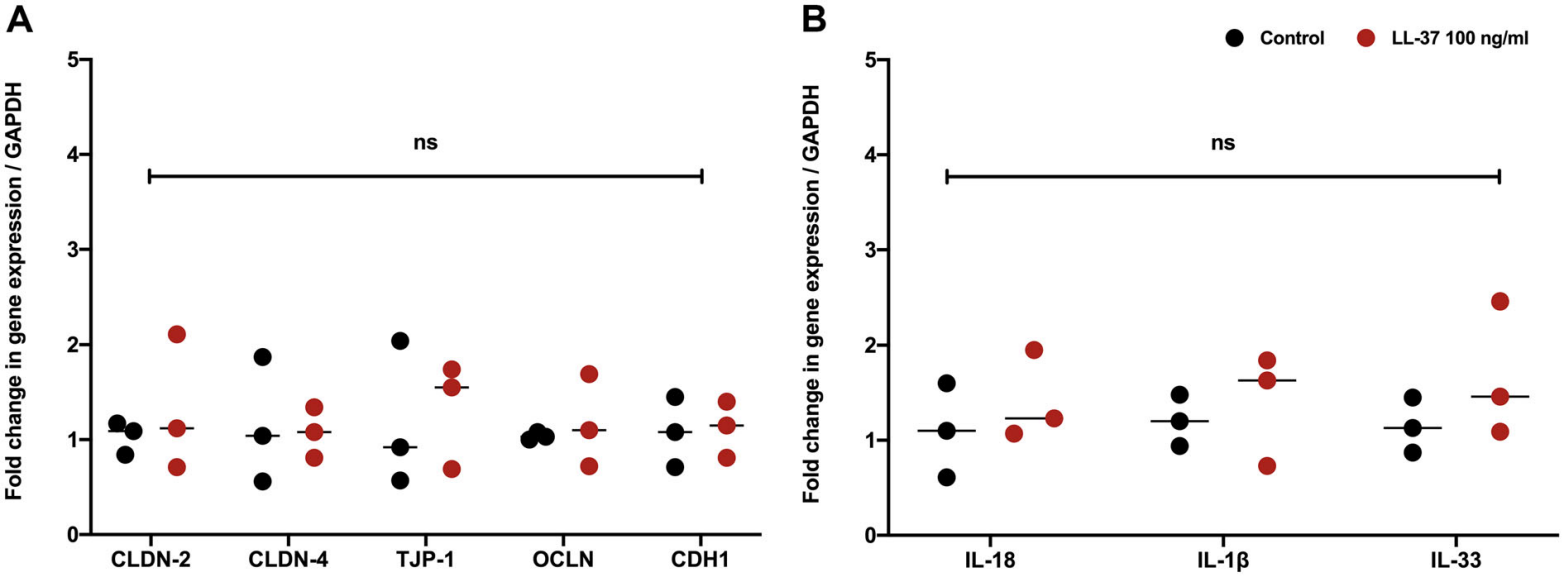
Recombinant LL‐37 stimulation of human colonic epithelial organoids does not impact expression of (A) key epithelial barrier permeability genes or (B) epithelium‐derived cytokine genes. Organoids did not express IL‐10, IL‐21, or IL‐27.

We next turned our attention to the functional consequences of LL‐37 on the colonic epithelial barrier. This was assessed using a human colon‐derived organoid culture with focus on the gene expression profile of key genes in the regulation of permeability and cytokine secretory capacity. The human colonic organoids did not express *IL‐10*, *IL‐22*, or *IL‐27*. Stimulation with LL‐37 did not significantly impact the expression of key barrier permeability or epithelial‐derived cytokine genes (Figure 3).

## Discussion

Cathelicidin exerts pleotropic effects depending on the tissue type [2, 3], and changes in expression are associated with cancer across different body sites [2]. Data on the expression profile of cathelicidin in human CRC are limited [13], and its biological impact in this pathology is unknown. We previously reported that loss of cathelicidin expression was associated with CRC in a pre‐clinical mouse model [5]. Here, we confirm translation of this pre‐clinical data to human disease and show that loss of LL‐37 expression is associated with human CRC in a large, robust, well‐characterised tissue cohort of human CRCs that is representative of this malignancy. This may indicate a tumour suppressor role of LL‐37 in CRC. This switch to reduced expression in the cytoplasm of epithelial cells occurs very early in the cancer process, with a significant reduction between non‐neoplastic mucosa and the earliest stages of CRC. Given that the expression of LL‐37 is not associated with survival, expression intensity of LL‐37 does not appear to be a prognostic biomarker to aid clinical management at the time of diagnosis. We did not assess whether LL‐37 expression is linked to treatment response in our study.

We next began to investigate how this dynamic LL‐37 expression profile impacts the biology of colorectal carcinogenesis. Our previous data revealed that the impact of cathelicidin in the gastrointestinal tract was linked to the colonic microbiota [5]. Dysbiosis of the microbiota is associated with CRC and there is evidence that some bacterial species such as *Fusobacterium* are enriched in CRC tissue [22, 23, 24] due to an increase in bacterial translocation across the epithelial barrier [25]. We hypothesised that LL‐37 may impact regulatory elements of barrier integrity and permeability, with loss of LL‐37 expression associated with increased permeability. In keeping with this hypothesis, Tatsuta *et al* demonstrated that LL‐37‐attenuated cigarette smoke induced epithelial damage in the respiratory tract through effects on regulation of epithelial permeability [26]. There are limited data on the effect of LL‐37 on the human gastrointestinal epithelial barrier and previous mechanistic studies used immortalised cell line models. Therefore, we assessed the impact of LL‐37 on permeability gene expression in a human colon tissue‐derived organoid model. Organoids are complex, three‐dimensional *ex vivo* tissue cultures and represent a near‐physiological model with advantages over traditional two‐dimensional immortalised cell cultures [27]. Permeability is regulated by an interacting network of different families of proteins located at the tight junction between epithelial cells, and examples include the claudins, occludin, and tight junctional proteins [28]. Here, we did not identify a change in expression of key mediators regulating epithelial barrier permeability and integrity in response to direct cathelicidin stimulation in our *ex vivo* human organoid model. This is in keeping with our previous pre‐clinical *in vivo* assessment of intestinal permeability, where there was no increase in serum FITC‐dextran in cathelicidin knockout mice and no change in expression of permeability genes in the colonic mucosa [5]. However, the focus of cathelicidin in the gut may be different depending on the context. As an example, in our previous studies in mouse models, knockout of the cathelicidin analogue CRAMP was associated with shorter crypt length, and this may indicate a role in epithelial cell proliferation in normal homeostasis [5]. Other changes were identified, such as changes in microbiota composition, and it is unclear if the LL‐37 effect on the epithelial barrier was direct or indirect. In contrast, in the murine model of CRC, loss of cathelicidin led to larger tumours suggesting a tumour suppressor role, and this latter effect is mirrored by our data here in a large cohort of human CRCs, and the data by Ren *et al* [13] in previous related studies. Therefore, the biological activities of cathelicidin in the gut may be different depending on the context and we need to consider this when interpreting the data from the organoid models. Our organoid models were derived from non‐neoplastic mucosa and our data reveal novel insights into cathelicidin biology in gut homeostasis, specifically on direct effects of LL‐37 on epithelial cell function. It may be that the cathelicidin effect will be different in organoid models derived from CRC and this requires further attention to define the biology of cathelicidin in the cancer context.

The immune response is one of the hallmarks of cancer [29, 30]. The tumour‐infiltrating lymphocyte phenotype is well established as a prognostic indicator in colorectal malignancy [31] and is the basis of the clinically applied immunoscore to aid with prognostication [32]. The presence of CD8^+^ T cells is associated with improved overall survival and cancer‐specific survival [33, 34]. Here, in CRC, CD8^+^ cell infiltrate is associated with increased intensity of epithelial LL‐37 expression from negative through weak and moderate expression. However, this association is less apparent in tumours that strongly express LL‐37. The reasons for this differential association and the biological consequences of this are unknown. We can speculate that the association may indicate a role of LL‐37 in microenvironmental changes that could impact pathogenesis, and appears most relevant in early colorectal neoplastic progression. It may be that strong expression of LL‐37 in the context of tumour could reflect a change in the functional status of the protein and this requires further investigation.

The relationship between LL‐37 expression and CD8^+^ T cell infiltrate was associated with mismatch repair protein status. CD8 lymphocytes play a key role in anti‐tumoural response in the cancer microenvironment, stimulating tumour cell apoptosis [35]. There is a well‐established link between mismatch repair protein status, immune cell infiltrate particularly CD8^+^ cells, expression of immune checkpoint inhibitors, and immune escape of the tumour in CRC, and these factors have been the focus of investigation into the predicted efficacy of immunotherapy in this subset of CRCs [36]. The link between mismatch repair protein status and CD8^+^ infiltrate is seen in other tumour types such as pancreatic cancer [37]. Our study does not explore whether LL‐37 could impact the function of CD8^+^ cells in CRC. However, this has been reported in other studies across different pathologies. As an example, LL‐37 is overexpressed in skin in psoriasis where it acts as an auto‐antigen for CD8^+^ T cells, leading to the up‐regulation of markers of cytotoxic granule release, cytokine release, and proliferation of these CD8^+^ cells, and contributing to disease pathogenesis [38]. Findlay *et al* [39] reported that LL‐37‐stimulated dendritic cells led to the proliferation, activation, and cytokine production by CD8^+^ cells. Furthermore, tumour antigen‐presenting LL‐37‐stimulated dendritic cells led to increased migration of activated CD8^+^ T cells into murine squamous cell carcinoma, resulting in tumour regression.

Overall, this study of a large robust cohort of 650 human CRC cases and a cutting‐edge human gastrointestinal epithelial organoid model reveals novel insights into the pathogenesis of CRC. We demonstrate that there is dynamic expression of LL‐37 in human CRC and this is linked to CD8^+^ T cell infiltrate, and that LL‐37 does not impact expression of human epithelial cell genes regulating permeability. Further work is required to assess the biological consequence of loss of LL‐37 expression in CRC to uncover novel treatment targets for this malignancy.

## Author contributions statement

RJP conducted experiments, analysed data, and prepared the initial draft of the paper. AA and SGC conducted experiments and analysed data. GIM provided pathology expertise in confirming tissue diagnosis on the tissue microarray and analysed data. MS‐T planned the experimental protocol for the immune cell phenotyping study and analysed data. MPH and JAJ contributed to data acquisition. TY and JMW helped to plan this study based on their murine model data, and analysed data. MHM provided the initial idea for the study, supervised experiments, analysed data, and prepared the paper. All authors reviewed the final paper and confirmed their approval for submission.

## Supporting information


Supplementary materials and methods
**Figure S1.** Epithelial expression of LL‐37 was not associated with survival in univariate or multivariate analysis
**Figure S2.** Densities of stromal CD3^+^, CD4^+^, and CD8^+^ T cells are associated with overall survival, which validates the TMA as representative of pathology
**Figure S3.** Epithelial cytoplasmic LL‐37 expression intensity is associated with density of stromal CD8^+^ T cells in CRC
**Table S1.** The association between epithelial cytoplasmic LL‐37 expression intensity and density of T cell infiltrate when stratified by different clinico‐pathological characteristics
