# Supplementary material for: Colonic epithelial cathelicidin (LL‐37) expression intensity is associated with progression of colorectal cancer and presence of CD8 + T cell infiltrate
Source: J Pathol Clin Res. 2021 May 14;7(5):495–506. doi: 10.1002/cjp2.222 (PMC8363930; doi:10.1002/cjp2.222)
Supplement: Supplementary file 1 — Supplementary materials and methods Figure S1. Epithelial expression of LL‐37 was not associated with survival in univariate or multivariate analysis Figure S2. Densities of stromal CD3+, CD4+, and CD8+ T cells are associated with overall survival, which validates the TMA as representative of pathology Figure S3. Epithelial cytoplasmic LL‐37 expression intensity is associated with density of stromal CD8+ T cells in CRC Table S1. The association between epithelial cytoplasmic LL‐37 expression intensity and density of T cell infiltrate when stratified by different clinico‐pathological characteristics [file CJP2-7-495-s001.docx]

**Colonic epithelial cathelicidin (LL-37) expression intensity is associated with progression of colorectal cancer and presence of CD8^+^ T-cell infiltrate**

RJ Porter *et al*. *J Pathol Clin Res* DOI: 10.1002/cjp2.222

**Supplementary material**

**Supplementary materials and methods**

**Figure S1.** Epithelial expression of LL-37 was not associated with survival with univariate or multivariate analysis

**Figure S2.** Densities of stromal CD3^+^, CD4^+^ and CD8^+^ T-cells are associated with overall survival, which validates the TMA as representative of pathology

**Figure S3.** Epithelial cytoplasmic LL-37 expression intensity is associated with density of stromal CD8^+^ T-cells in colorectal cancer

**Table S1.** The association between epithelial cytoplasmic LL-37 expression intensity and density of T-cell infiltrate when stratified by different clinico-pathological characteristics.

**Supplementary material and methods**

**Colonic Organoid Tissue Culture Media**

1. **Initiation Media**

1% Bovine Serum Albumin, 2 mM Glutamax, 2 mM L-glutamine, 10 mM HEPES, 1X N2, 1X B27, 1 mM N-acetylcysteine, 50 ng/mL EGF, 10 mM nicotinamide, 500 nM A83-01, 10 nM prostaglandin E2, 10 nM [Leu-15]-gastrin1, 10 uM SB202190, 2.5 uM Thiazovinin, 10 uM Y-27632 dihydrochloride, 2.5 uM CHIR99021 and 100 μg/mL Primocin.

1. **Maintenance Media**

1% Bovine Serum Albumin, 2 mM Glutamax, 2 mM L-glutamine, 10 mM HEPES, 1X N2, 1X B27, 1 mM N-acetylcysteine, 50 ng/mL EGF, 10 mM nicotinamide, 500 nM A83-01, 10 nM prostaglandin E2, 10 nM [Leu-15]-gastrin1, 10 uM SB202190, 2.5 uM Thiazovinin, and 100 μg/mL Primocin.

1. **Differentiation Media**

1% Bovine Serum Albumin, 100 ng/mL Wnt3A, 500 ng/mL R-spondin-1, 100 ng/mL noggin, 2 mM Glutamax, 2 mM L-glutamine, 10 mM HEPES, 1X N2, 1X B27, 1 mM N-acetylcysteine, 50 ng/mL EGF, 500 nM A83-01, 10 nM prostaglandin E2, 10 nM [Leu-15]-gastrin1, 2.5 uM Thiazovinin, and 100 μg/mL Primocin.


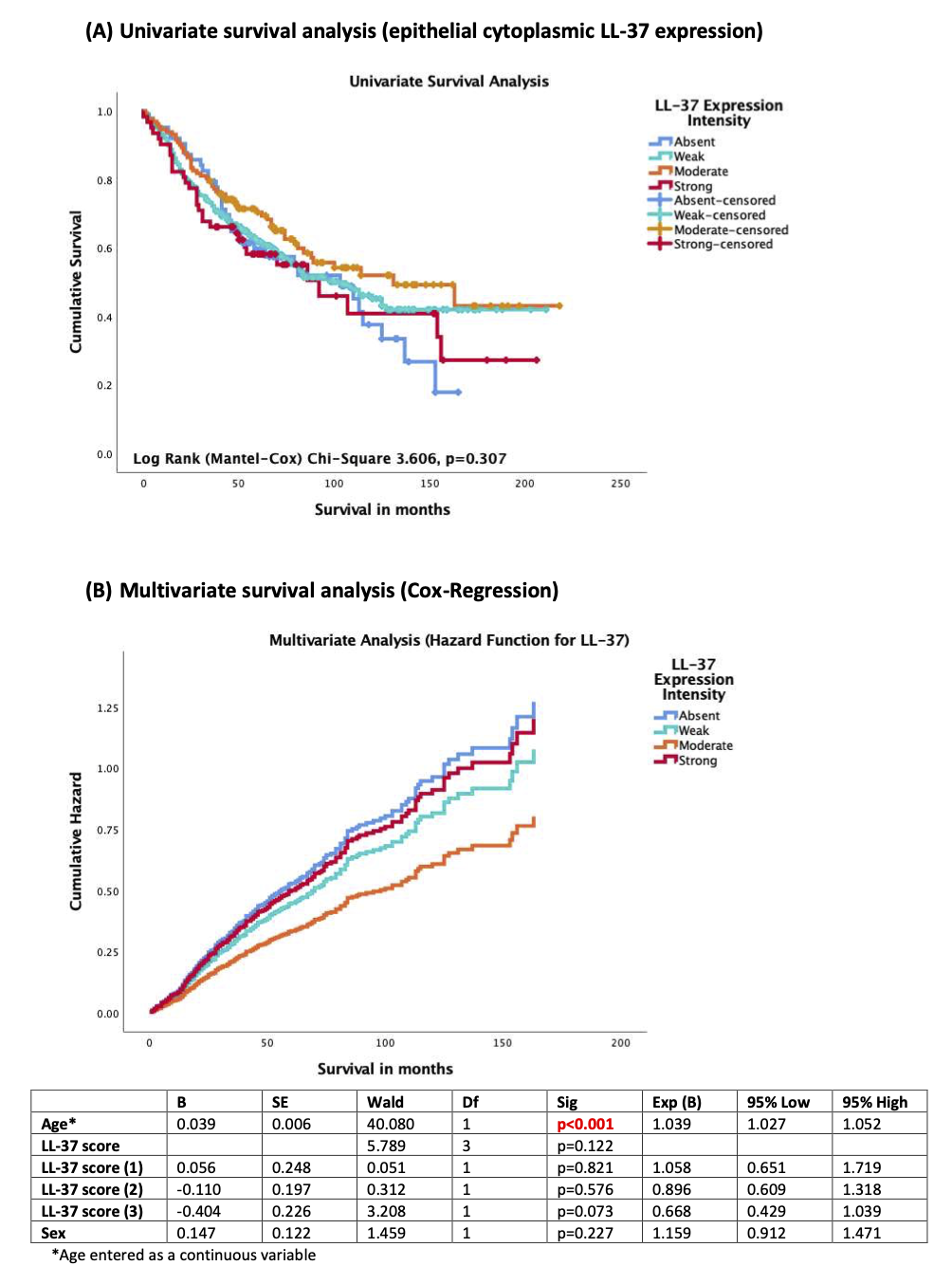


**Figure S1.** Epithelial expression of LL-37 was not associated with survival with univariate or multivariate analysis


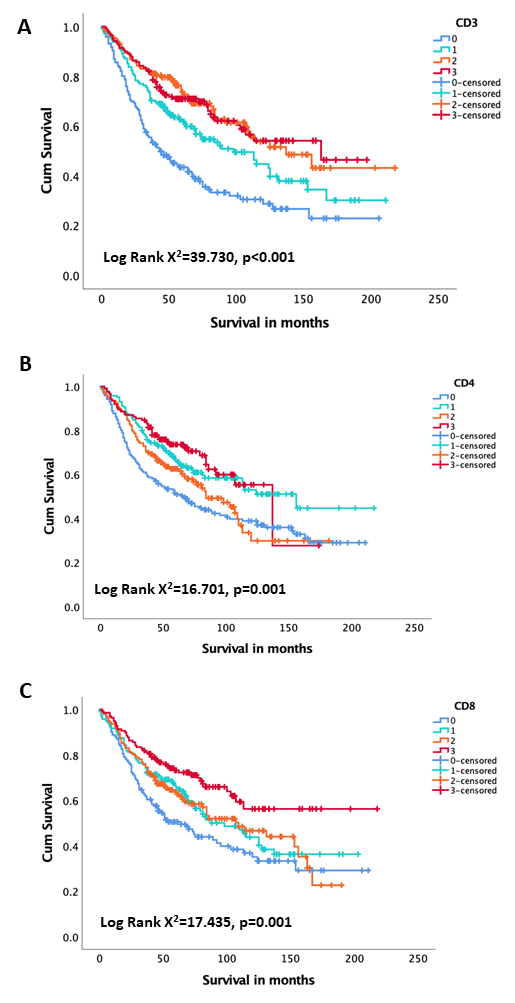


**Figure S2.** Densities of stromal (A) CD3^+^, (B) CD4^+^ and (C) CD8^+^ T-cells are associated with overall survival, which validates the TMA as representative of pathology. 0,1,2,3 represent 0-25%, 26-50%, 51-75%, 76-100% density as a percentage of total immune cell infiltrate respectively. Analysis with Kaplan Meier Log Rank (Mantex-Cox) tests.


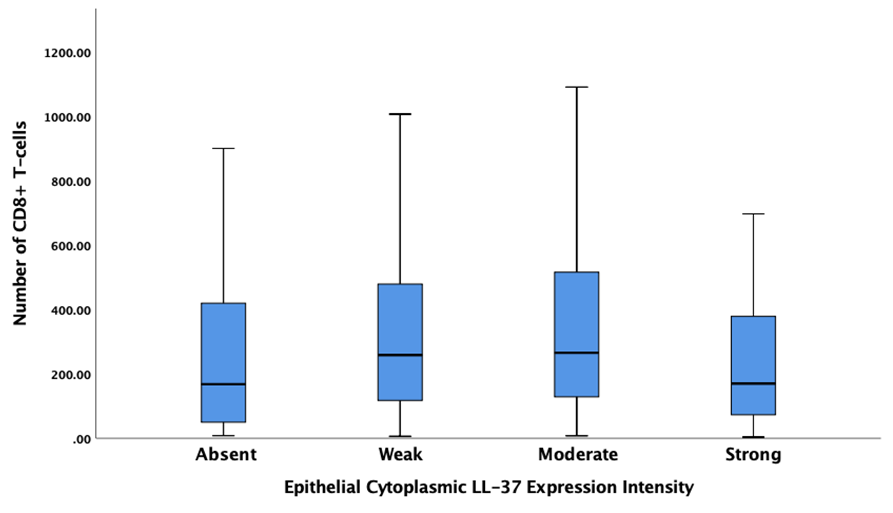


**Figure S3.** Epithelial cytoplasmic LL-37 expression intensity is associated with density of stromal CD8^+^ T-cells in colorectal cancer. Absent (215.43), weak (275.45), moderate (285.33) and strong (220.98) mean ranks suggest increased expression from absent through moderate, then reduced expression with strong LL-37 expression. Analysis with Kruskal-Wallis test.

**Table S1A-F. T**hese tables present the data for the association between epithelial cytoplasmic LL-37 expression intensity and density of T-cell infiltrate when stratified by different clinico-pathological characteristics. Analysed by Kruskal-Wallis (KW) or Mann-Whitney U (MWU) tests, as appropriate. Bold values indicate p<0.05

**Table S1A. Association between epithelial cytoplasmic LL-37 expression intensity and density of stromal T-cells (unstratified).**

|  | **Absent v weak v moderate v strong** | | **Absent v weak, moderate and strong** | | **Absent and weak v moderate and strong** | | **Strong v absent, weak and moderate** | |
| --- | --- | --- | --- | --- | --- | --- | --- | --- |
|  | **KW** | **p** | **MWU** | **p** | **MWU** | **p** | **MWU** | **p** |
| CD3^+^ T-cells | 4.222 | 0.238 | 15525.500 | 0.510 | 32359.000 | 0.903 | 11957.000 | 0.083 |
| CD4^+^ T-cells | 5.175 | 0.159 | 16201.500 | 0.101 | 33828.500 | 0.292 | 12466.000 | 0.455 |
| CD8^+^ T-cells | 14.197 | **0.003** | 17104.500 | **0.007** | 31231.000 | 0.962 | 10281.000 | **0.026** |

**Table S1B. Association between epithelial cytoplasmic LL-37 expression intensity and density of stromal T-cells, stratified by UICC stage.**

|  | **Absent v weak v moderate v strong** | | **Absent v weak, moderate and strong** | | **Absent and weak v moderate and strong** | | **Strong v absent, weak and moderate** | |
| --- | --- | --- | --- | --- | --- | --- | --- | --- |
|  | **KW** | **p** | **MWU** | **p** | **MWU** | **p** | **MWU** | **p** |
| **UICC Stage 1** | | | | | | | | |
| CD3^+^ T-cells | 0.810 | 0.847 | 401.000 | 0.936 | 1586.000 | 0.582 | 671.000 | 0.718 |
| CD4^+^ T-cells | 3.643 | 0.303 | 494.000 | 0.270 | 1728.500 | 0.074 | 733.500 | 0.490 |
| CD8^+^ T-cells | 5.607 | 0.132 | 471.000 | 0.501 | 1680.000 | 0.358 | 583.000 | 0.137 |
| **UICC Stage 2** | | | | | | | | |
| CD3^+^ T-cells | 0.262 | 0.967 | 2752.000 | 0.628 | 4901.500 | 0.807 | 1769.000 | 0.984 |
| CD4^+^ T-cells | 2.831 | 0.418 | 2795.000 | 0.156 | 4606.000 | 0.673 | 1530.000 | 0.547 |
| CD8^+^ T-cells | 3.728 | 0.292 | 2901.500 | 0.072 | 4363.000 | 0.834 | 1519.000 | 0.637 |
| **UICC Stage 3** | | | | | | | | |
| CD3^+^ T-cells | 7.312 | 0.063 | 2479.000 | 0.923 | 4254.000 | 0.074 | 1589.000 | **0.009** |
| CD4^+^ T-cells | 2.000 | 0.572 | 2614.000 | 0.742 | 5164.500 | 0.718 | 1948.500 | 0.194 |
| CD8^+^ T-cells | 10.342 | **0.016** | 2905.000 | 0.060 | 4369.500 | 0.177 | 1300.000 | **0.022** |

**Table S1C. Association between epithelial cytoplasmic LL-37 expression intensity and density of stromal T-cells, stratified by location.**

|  | **Absent v weak v moderate v strong** | | **Absent v weak, moderate and strong** | | **Absent and weak v moderate and strong** | | **Strong v absent, weak and moderate** | |
| --- | --- | --- | --- | --- | --- | --- | --- | --- |
|  | **KW** | **p** | **MWU** | **p** | **MWU** | **p** | **MWU** | **p** |
| **Colon** | | | | | | | | |
| CD3^+^ T-cells | 1.820 | 0.611 | 8152.500 | 0.645 | 19304.000 | 0.757 | 8034.000 | 0.236 |
| CD4^+^ T-cells | 5.878 | 0.118 | 8658.500 | 0.095 | 20856.000 | 0.100 | 8280.500 | 0.833 |
| CD8^+^ T-cells | 10.917 | **0.012** | 9330.000 | **0.012** | 18671.000 | 0.876 | 6723.000 | 0.082 |
| **Rectum** | | | | | | | | |
| CD3^+^ T-cells | 3.174 | 0.366 | 1128.000 | 0.502 | 1676.500 | 0.736 | 347.000 | 0.177 |
| CD4^+^ T-cells | 2.068 | 0.558 | 1153.000 | 0.488 | 1572.500 | 0.500 | 411.500 | 0.291 |
| CD8^+^ T-cells | 4.095 | 0.251 | 1143.000 | 0.245 | 1622.000 | 0.922 | 356.000 | 0.135 |

**Table S1D. Association between epithelial cytoplasmic LL-37 expression intensity and density of stromal T-cells, stratified by degree of tumour differentiation.**

|  | **Absent v weak v moderate v strong** | | **Absent v weak, moderate and strong** | | **Absent and weak v moderate and strong** | | **Strong v absent, weak and moderate** | |
| --- | --- | --- | --- | --- | --- | --- | --- | --- |
|  | **KW** | **p** | **MWU** | **p** | **MWU** | **p** | **MWU** | **p** |
| **Poor Differentiation** | | | | | | | | |
| CD3^+^ T-cells | 3.771 | 0.287 | 18.000 | 0.403 | 130.000 | 0.896 | 41.000 | 0.183 |
| CD4^+^ T-cells | 4.789 | 0.188 | 35.000 | 0.795 | 185.000 | 0.073 | 65.000 | 0.827 |
| CD8^+^ T-cells | 5.368 | 0.147 | 16.000 | 0.348 | 107.000 | 0.653 | 19.000 | **0.039** |
| **Moderate-to-well Differentiation** | | | | | | | | |
| CD3^+^ T-cells | 3.112 | 0.375 | 14383.500 | 0.355 | 28269.000 | 0.870 | 10628.000 | 0.180 |
| CD4^+^ T-cells | 3.900 | 0.273 | 14691.000 | 0.098 | 28880.500 | 0.511 | 10781.500 | 0.545 |
| CD8^+^ T-cells | 13.023 | **0.005** | 15834.500 | **0.003** | 27419.500 | 0.935 | 9407.000 | 0.091 |

**Table S1E. Association between epithelial cytoplasmic LL-37 expression intensity and density of stromal T-cells, stratified by extramural vascular invasion (EMVI).**

|  | **Absent v weak v moderate v strong** | | **Absent v weak, moderate and strong** | | **Absent and weak v moderate and strong** | | **Strong v absent, weak and moderate** | |
| --- | --- | --- | --- | --- | --- | --- | --- | --- |
|  | **KW** | **p** | **MWU** | **p** | **MWU** | **p** | **MWU** | **p** |
| **No EMVI** | | | | | | | | |
| CD3^+^ T-cells | 2.256 | 0.521 | 10249.000 | 0.662 | 20616.000 | 0.876 | 7339.000 | 0.187 |
| CD4^+^ T-cells | 3.837 | 0.280 | 10805.000 | 0.107 | 21197.500 | 0.352 | 7495.000 | 0.652 |
| CD8^+^ T-cells | 10.390 | **0.016** | 11355.500 | **0.038** | 20199.000 | 0.971 | 6404.500 | **0.034** |
| **EMVI** | | | | | | | | |
| CD3^+^ T-cells | 1.878 | 0.598 | 539.000 | 0.451 | 1212.000 | 0.592 | 572.000 | 0.292 |
| CD4^+^ T-cells | 1.336 | 0.721 | 570.000 | 0.333 | 1436.000 | 0.718 | 673.500 | 0.774 |
| CD8^+^ T-cells | 6.579 | 0.087 | 602.000 | **0.015** | 1169.500 | 0.953 | 475.000 | 0.587 |

**Table S1F. Association between epithelial cytoplasmic LL-37 expression intensity and density of stromal T-cells, stratified by microsatellite instability (MSI) status.**

|  | **Absent v weak v moderate v strong** | | **Absent v weak, moderate and strong** | | **Absent and weak v moderate and strong** | | **Strong v absent, weak and moderate** | |
| --- | --- | --- | --- | --- | --- | --- | --- | --- |
|  | **KW** | **p** | **MWU** | **p** | **MWU** | **p** | **MWU** | **p** |
| **MSI Proficient** | | | | | | | | |
| CD3^+^ T-cells | 3.526 | 0.317 | 11440.500 | 0.654 | 23302.500 | 0.527 | 9403.000 | 0.081 |
| CD4^+^ T-cells | 4.440 | 0.218 | 11686.000 | 0.285 | 25123.000 | 0.357 | 9808.500 | 0.321 |
| CD8^+^ T-cells | 13.298 | **0.004** | 12914.500 | **0.007** | 22623.500 | 0.717 | 8308.000 | **0.036** |
| **MSI Deficient** | | | | | | | | |
| CD3^+^ T-cells | 2.916 | 0.405 | 232.000 | 0.652 | 595.000 | 0.240 | 98.000 | 0.482 |
| CD4^+^ T-cells | 4.014 | 0.260 | 295.000 | 0.052 | 492.000 | 0.902 | 86.000 | 0.848 |
| CD8^+^ T-cells | 2.296 | 0.513 | 223.000 | 0.731 | 557.5000 | 0.397 | 64.000 | 0.373 |
